# Efficacy and Safety of Abciximab in Diabetic Patients Who Underwent Percutaneous Coronary Intervention with Thienopyridines Loading: A Meta-Analysis

**DOI:** 10.1371/journal.pone.0020759

**Published:** 2011-06-03

**Authors:** Yihua Wu, Yu Shi, Han Wu, Chang Bian, Qian Tang, Geng Xu, Jun Yang

**Affiliations:** 1 Department of Cardiology, The Second Affiliated Hospital, Zhejiang University School of Medicine, Hangzhou, Zhejiang, China; 2 State Key Laboratory for Diagnosis and Treatment of Infectious Diseases, The First Affiliated Hospital, Zhejiang University School of Medicine, Hangzhou, Zhejiang, China; 3 Department of Ophthalmology, The Second Affiliated Hospital, Zhejiang University School of Medicine, Hangzhou, Zhejiang, China; 4 Department of Toxicology, Hangzhou Normal University School of Public Health, Hangzhou, Zhejiang, China; University of Tor Vergata, Italy

## Abstract

**Background:**

It has been controversial whether abciximab offered additional benefits for diabetic patients who underwent percutaneous coronary intervention (PCI) with thienopyridines loading.

**Methods:**

MEDLINE, EMBASE, the Cochrane library clinical trials registry, ISI Science Citation Index, ISI Web of Knowledge and China National Knowledge Infrastructure (CNKI) were searched, supplemented with manual-screening for relevant publications. Quantitative meta-analyses were performed to assess differences between abciximab groups and controls with respect to post-PCI risk of major cardiac events (MACEs), angiographic restenosis and bleeding complications.

**Results:**

9 trials were identified, involving 2,607 diabetic patients receiving PCI for coronary artery diseases. Among those patients who underwent elective PCI or primary PCI, pooling results showed that abciximab did not significantly reduce risks of MACEs (for elective-PCI patients: RR_1-month_: 0.93, 95% CI: 0.60–1.44; RR_1-year_: 0.95, 95% CI: 0.81–1.11; for primary-PCI patients: RR_1-month_: 1.05, 95% CI: 0.70–1.57; RR_1-year_: 0.98, 95% CI: 0.80–1.21), nor all-cause mortality, re-infarction and angiographic restenosis in either group. The only beneficial effect by abciximab appeared to be a decrease 1-year TLR (target lesion revascularization) risk in elective-PCI patients (RR1-year: 0.83, 95% CI: 0.70–0.99). Moreover, occurrence of minor bleeding complications increased in elective-PCI patients treated with abciximab (RR: 2.94, 95% CI: 1.68–5.13, *P*<0.001), whereas major bleedings rate was similar (RR: 0.83, 95% CI: 0.27–2.57).

**Conclusions:**

Concomitant dosing of abciximab and thienopyridines provides no additional benefit among diabetic patients who underwent PCI; this conclusion, though, needs further confirmation in larger studies.

## Introduction

Percutaneous coronary intervention (PCI) has been shown to significantly reduce the prevalence of major cardiac events (MACEs) in patients with myocardial infarction, while diabetic patients represent a distinct sub-population at higher risk of developing MACEs or restenosis after PCI than non-diabetic population. Thus, it is plausible that diabetic patients might benefit from a more aggressive anti-platelet aggregation therapy. [1], [2], [3], [4], [5].

Anti-platelet therapy is an important adjunctive treatment that reduces ischaemic complications in patients who underwent PCI [6], [7], [8], [9]. Two anti-platelet drugs, Thienopyridine (clopidogrel or ticlopidine) and the platelet glycoprotein IIb/IIIa inhibitor (abciximab) were conditionally recommended in coronary artery disease (CAD) patients who underwent PCI in recently updated guidelines [9], [10], [11].

Abciximab, a platelet glycoprotein IIb/IIIa inhibitor, acts by competing with the ligand (e.g., fibrinogen) binding that is essential for platelet bridging and aggregate formation [12], [13], [14], [15], [16], [17]. Abciximab has been demonstrated in several studies [18], [19], [20] to improve outcomes in diabetic patients treated with PCI; these conclusion have been supported also by a meta-analysis in 2003 [20]. In recent years, however, thienopyridines loading has been widely used before PCI to prevent MACEs and restenosis, according to current practice guidelines as recommendation class level I (“Should be performed/administered”) [9], [11], [21], [22], and thus, whether addition of abciximab is beneficial became elusive.

Thienopyridine (clopidogrel and ticlopidine) is a classic anti-platelet agent, which is considered to be a significant advancement for PCI [23], [24], [25]. Thienopyridine inhibits ADP-induced platelet aggregation and signaling pathways within the platelet, which is different from the anti-platelet mechanism elicited by GPIIb/IIIa inhibitors such as abciximab [12], [26], [27], [28], suggesting a possibility for combination therapy, especially among the high-risk patients who need stent implantation. Such combination a regimen was also conditionally recommended in newly published guidelines [29], [30], [31], [32], [33]. Some other investigators, however, have found evidence to support the opposite view [34], [35], [36].

To address this issue, we carried out a systematic review and meta-analysis to re-evaluate whether adjuvant therapy with abciximab is necessary in diabetic patients who underwent PCI with a loading dose of thienopyridine.

## Methods

### Data sources, search strategy, and selection criteria

Systematic literature searches were conducted in six databases: Medline, the Cochrane Library, EMBASE, ISI Science Citation Index, ISI Web of Knowledge, and China National Knowledge Infrastructure (CNKI) in July, 2010. The search term “abciximab” was used in combination with “diabetes” and “PCI”. The “references” sections of reviews and original articles were also scanned for missing trials.

We included articles if they met all the following criteria: (1) diabetic patients who underwent PCI were included, and types of coronary artery diseases were stratified clearly; (2) Diabetes mellitus was diagnosed according to the World Health Organization criteria [37]; (3) a loading dose of thienopyridine (clopidogrel or ticlopidine) should be adopted, and the same protocol of abciximab-therapy were adopted in all trials (a bolus of 0.25 mg/kg, a 12-h infusion (0.125 µg/kg/min)); (4) primary endpoints of interest were the composite incidence of MACEs, the secondary end point of interest was the frequency of angiographic restenosis, which were described in Endpoints and definition; (5) a follow-up duration ≥1-month; and (6) the study was designed as randomized clinical trial.

Two reviewers (Wu YH, MD, PhD and Shi Y, PhD) independently extracted study characteristics using standardized forms. A total of 12 articles were identified, including 9 randomized control trials (RCTs) such as DANTE, ISAR-SWEET, ISAR-REACT, ISAR-REACT2, ASIAD, CADILLAC, BRAVE-3, and two conducted by Deluca [34], [35], [36], [38], [39], [40], [41], [42], [43], [44], [45], [46], that were suitable for inclusion in the meta-analysis. Besides the published data, the unpublished original data of diabetic patients were extracted for analysis from ISAR-SWEET, ISAR-REACT, ISAR-REACT2 and BRAVE3 data provided by Drs. Kastrati and Mehilli (Deutsches Herzzentrum, Munich, Germany). All these studies were high-quality RCTs (Dephi ≥6) [47]. The process of selection was shown in Table S3 and Table S4.

For each trial, results at short-term (1-month) or long-term (6–12 months) follow-up were extracted for this analysis, and the data were retrieved according to the intention-to-treat principle. Restrictions with respect to language or publication date were not placed on these searches.

### Endpoints and definitions

The primary endpoint of interest was the composite incidence of MACEs at 1-month and 1-year. MACEs were a composite of death of any cause, non-fatal re-infarction, and any repeated intervention or revascularization of the target vessel as a result of ischemia. During the follow-up period, re-infarction was defined as new onset of ischemic symptoms or ischemic changes on electrocardiography with over 2-fold normal total creatine kinase value or new pathological Q-waves. Target lesion revascularization (TLR) was defined as clinically driven percutaneous revascularization or bypass of the target lesion or any segment of the epicardial coronary artery containing the target lesion. The frequency of angiographic restenosis (diameter stenosis >50%) was the secondary endpoint of interest. Bleeding events were defined as major or minor according to the thrombolysis in myocardial infarction (TIMI) classification[48].

### Data synthesis and Statistical analyses

Quantitative meta-analysis was carried out using Cochrane Review Manager (Revman) software 5.0. Results were generated using endpoint values for supplement minus control group, and summarized as Forest plots. Relative risks (RRs) with 95% confidence intervals (CIs) were used to assess the comparative effect of abciximab *versus* control in diabetic patients treated by PCI. A fixed-effect model with the Mantel–Haenszel method was used to pool these RRs. The extent of heterogeneity across studies was checked using the test and *I* ^2^ test (*I* ^2^ test quantifies the proportion of total variation across studies due to heterogeneity rather than chance). A *P* value ≤0.10 in combination with an *I* ^2^>50% indicates significant heterogeneity, and the random-effect model was used if heterogeneity was present.

The continuous data are expressed as mean value ± SD. The categorical data are presented as counts or proportions. The differences between Abciximab and Placebo groups were assessed by χ^2^ test in diabetic patients with 600 mg clopidogrel loading (data from the three ISAR trials and BRAVE-3 trial). Sensitivity analyses were carried out to assess the stability of the overall estimates with regard to the use of PCI. A two-tailed *P* value that was less than 0.05 was considered to represent a significant variation alone. To assess for publication bias, funnel plots (i.e., plots of study results against precision) were constructed. The Egger's regression test was adopted to test the asymmetry of funnel plots using STATA software (version 10.0, StataCorp, College Station, TX, USA). A *P* value <0.10 was considered significant [49], [50].

We performed an “effect model analysis” [51] to investigate whether the effect of abciximab was dependent on the baseline risk of the studied population using SPSS software (SPSS Inc; Chicago, version 16.0). Briefly, three possibilities occurred, as described by Dr. Corvol [52].

## Results

### 1. Search results

**Figure 1:**
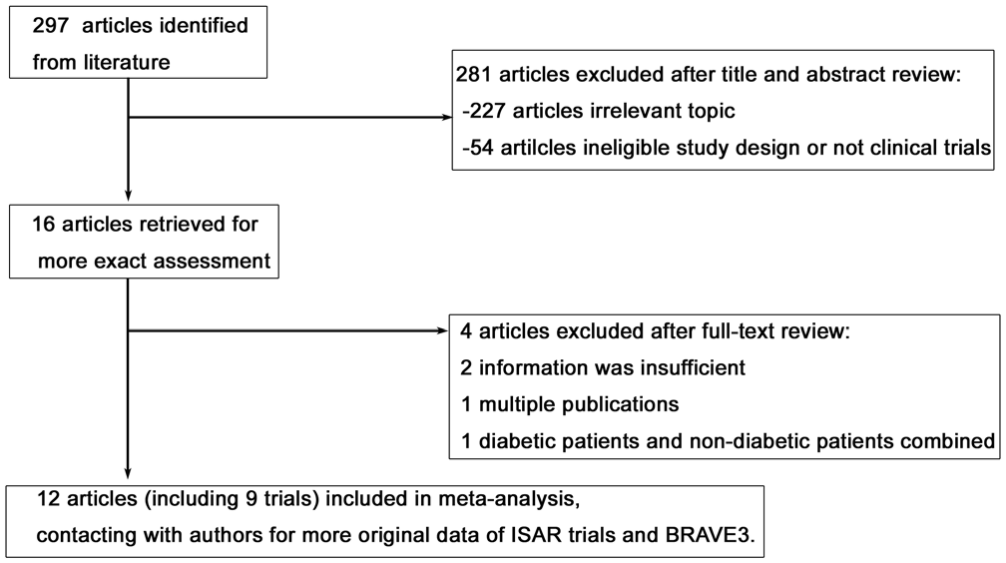
Selection of studies included in systematic review.

After searching Medline, the Cochrane Library, EMBASE, ISI Science Citation Index, ISI Web of Knowledge, and CNKI, we identified 297 abstracts which were then reviewed for inclusion and exclusion criteria (Figure 1 and Table S3). Review of abstracts and titles resulted in exclusion of 281 reports, and the remaining 16 articles were extracted for further assessment. After full-text review, 4 of the remaining 16 articles were excluded because the information from 2 papers was insufficient, 1 was a duplicate publication, and 1 was not the right subject for our review. The remaining 12 articles (including 9 the separate trials listed in Table S1; 1-month and 1-year outcomes of ISAR-REACT, ISAR-REACT2, and BRAVE3 trials were published multiple articles) were included in our study, then we contacted with authors and analyzed the original data of diabetic patients. Original data of diabetic arms of ISAR-SWEET, ISAR-REACT, ISAR-REACT2 and BRAVE-3 were provided by Drs. Kastrati and Mehilli. We also checked our meta-analysis according to the MOOSE guidelines, see Table S4.

### 2. Characteristics of included trials

The characteristics of the included studies (a total of 2607 diabetics) were summarized in Table S1 and Table S2. All articles were published between 2004 and 2010. Seven trials were performed in Europe and US [34], [39], [40], [42], [43], [44], [45], [46], [35], [36], one in Asia [41], and one in Brazil [38]. Among these studies, the diabetic patients in the abciximab and placebo/inactive control arms were included in our meta-analysis (n = 2607). In three trials (5 articles were included), only AMI patients who underwent primary PCI were included [42], [43], [44], [45], [46], while non-AMI patients undergoing elective PCI were investigated in the remaining trials. In four studies, diabetic patients were restricted to Type2 diabetes mellitus [34], [38], [40], [41], while the remaining studies included diabetic patients diagnosed according to the World Health Organization criteria [37]. The mean follow-up duration was 8.6 months and ranged from 1 month to 12 months. Four of the 9 trials used a high loading dose of 600 mg clopidogrel [34], [39], [40], [43], [44], [45], [46], and the remaining used 300 mg clopidogrel or 500 mg Ticlopidine. All studies used the same dose of abciximab (a bolus of 0.25 mg/kg, a 12-h infusion (0.125 g/kg/min)).

Overall studies included were assessed according to the Dephi criteria, and all of 9 trials were deemed high quality (≥6) as shown in Table S1. All trials were double-blind trials except DANTE trial [38].

### 3. Baseline patient characteristics

The 9 trials included a total of 2607 diabetic patients randomly assigned to abciximab (1317) *versus* placebo/control (1290) groups. Baseline characteristics of diabetics with elective and primary PCI were listed separately in Table S2. The mean ages ranged from 59 to 66 and 68% of the patients were male. Patients who were treated by insulin ranged from 1% to 43%. More than 50% patients had hypertension and hyperlipidemia. Patients who had previous myocardial infarction ranged from 10% to 67%, and 65% of the patients suffered from multivessel diseases. 95% of the patients finally underwent stent implantation.

### 4. Efficacy and safety of abciximab in diabetic patients undergoing elective PCI and primary PCI

#### 4.1 Mortality (1-month and 1-year)

**Figure 2:**
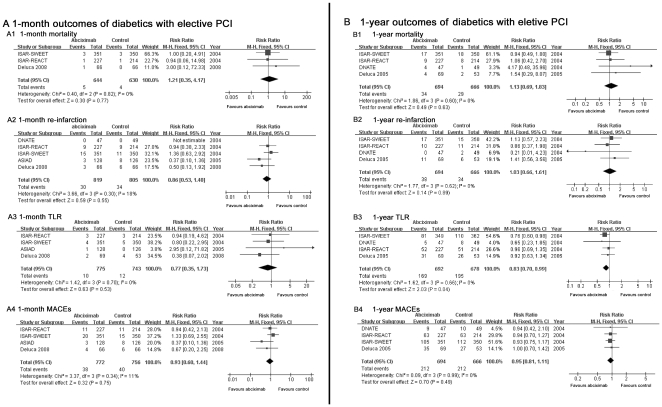
Comparison of 1-month and 1-year outcomes between abciximab and control group in patients with elective PCI. Efficacy of abciximab compared with control for (A1): 1-month mortality and (B1): 1-year mortality; (A2): 1-month reinfarction and (B2): 1-year reinfarction; (A3): 1-month TLR and (B3): 1-year TLR; (A4): 1-month MACEs and (B4): 1-year MACEs in diabetic patients with elective PCI.

**Figure 3:**
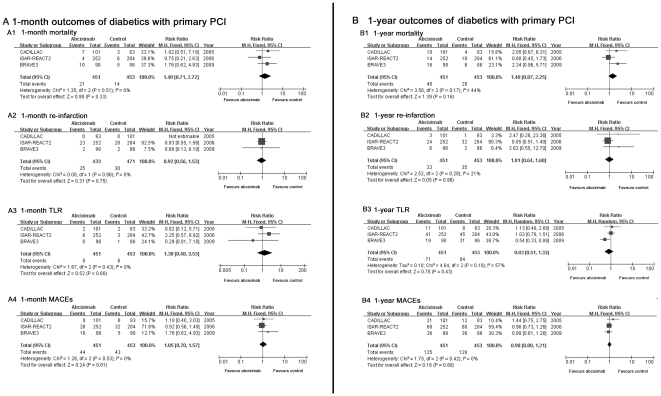
Comparison of 1-month and 1-year outcomes between abciximab and control group in patients with primary PCI. Efficacy of abciximab compared with control for (A1): 1-month mortality and (B1): 1-year mortality; (A2): 1-month reinfarction and (B2): 1-year reinfarction; (A3): 1-month TLR and (B3): 1-year TLR; (A4): 1-month MACEs and (B4):1-year MACEs in diabetic patients with primary PCI.

Pooled results showed that there was no significant difference in mortality between the abciximab group and non-abciximab group in diabetics with elective PCI at either 1-month (RR = 1.21, 95% CI: 0.35–4.17, *P* = 0.77, see Figure 2A1) or 1-year follow-up (RR = 1.13, 95% CI: 0.69–1.83, *P* = 0.63, see Figure 2B1). Similarly, abciximab did not reduce 1-month (RR = 1.40, 95% CI: 0.71–2.72, *P* = 0.33, see Figure 3A1) or 1-year mortality (RR = 1.40, 95% CI: 0.87–2.25, *P* = 0.16, see Figure 3B1) in diabetics with primary PCI.

#### 4.2 Re-infarction (1-month and 1-year)

The 1-month (Elective-PCI: RR = 0.86, 95% CI: 0.53–1.40, *P* = 0.55, Figure 2A2; Primary-PCI: RR = 0.92, 95% CI: 0.56–1.53, *P* = 0.75, Figure 3A2) and 1-year risk (Elective-PCI: RR = 1.03, 95% CI: 0.66–1.61, *P* = 0.89, Figure 2B2; Primary-PCI: RR = 1.01, 95% CI: 0.64–1.60, *P* = 0.96, Figure 3B2) for re-infarction showed no difference between abciximab group and control group in either primary-PCI or elective-PCI patients.

#### 4.3 TLR (1-month and 1-year)

For elective-PCI patients with abciximab, there was a moderate reduction of risk in TLR at 1-year follow-up (RR = 0.83, 95% CI = 0.70–0.99, *P* = 0.04, see Figure 2B3) but not at 1-month follow-up (RR = 0.77, 95% CI = 0.35–1.73, *P* = 0.53, see Figure 2A3). This protective effect by abciximab, however, did not appear to be significant at 1-month (RR: 1.30, 95%CI: 0.48–3.53, *P* = 0.60, Figure 3A3) or 1-year follow-up (RR: 0.83, 95% CI: 0.51–1.33, *P = *0.43, Figure B3) in patients receiving primary PCI.

#### 4.4 MACEs (1-month and 1-year)

Our results showed that elective-PCI patients treated with abciximab did not display a significant reduction in risk for MACEs as compared with those received placebo at either 1-month (RR: 0.93, 95% CI: 0.60–1.44, *P* = 0.75, see Figure 2A4) or 1-year follow-up (RR: 0.95, 95% CI: 0.81–1.11, *P* = 0.49, see Figure 2B4). Nor was any significant risk reduction by abciximab observed in patients undergoing primary PCI at either 1-month (RR: 1.05, 95% CI: 0.70–1.57, *P* = 0.81, see Figure 3A4) or 1-year follow-up (RR: 0.98, 95% CI: 0.80–1.21, *P* = 0.88, see Figure B4).

### 5. Angiographic restenosis (6-month)

**Figure 4:**
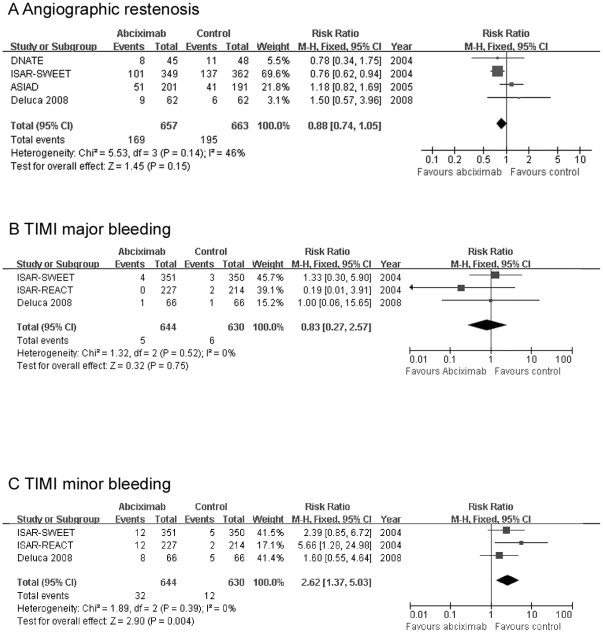
Comparison of secondary endpoints between abciximab and control group in patients with elective PCI. Efficacy of abciximab compared with control for (A) angiographic restenosis (B) TIMI major bleeding in diabetic patients with elective PCI; (C) TIMI minor bleeding in diabetic patients with elective PCI.

Information on angiographic restenosis was available in 4 trials [36], [38], [39], [41] for patients undergoing elective PCI. No significant difference in the rate of angiographic restenosis was observed between the two groups (RR: 0.88, 95% CI: 0.74–1.05, *P* = 0.15, see Figure 4A).

No trials evaluating primary-PCI patients reported this endpoint.

### 6. Bleeding complications (in-hospital)

Abciximab significantly aggravated TIMI minor bleedings (RR: 2.62, 95% CI: 1.37–5.03, *P* = 0.004; see Figure C) but not major bleedings (RR: 0.83, 95% CI: 0.27–2.57, *P* = 0.75; see Figure 4B) in patients with elective PCI.

Two trials reported (ISAR-REACT2 and BRAVE3) bleeding complications for ACS patients with primary PCI. The result of ISAR-REACT2 showed that abciximab significantly increased the risk of TIMI major bleeding in primary-PCI patients (RR = 10.14, *P* = 0.006). A similar trend was also observed in BRAVES trial, but was not statistically significant (RR = 2.63, *P* = 0.38). As to minor bleeding complications, the result from ISAR-REACT2 suggested that abciximab might aggravate TIMI minor bleeding (RR = 4.12, *P* = 0.007), which was not consistent with the result of BRAVE3 (RR = 0.88, *P* = 0.89).

### 7. Publication bias and Assessment of within-group heterogeneity

**Figure 5:**
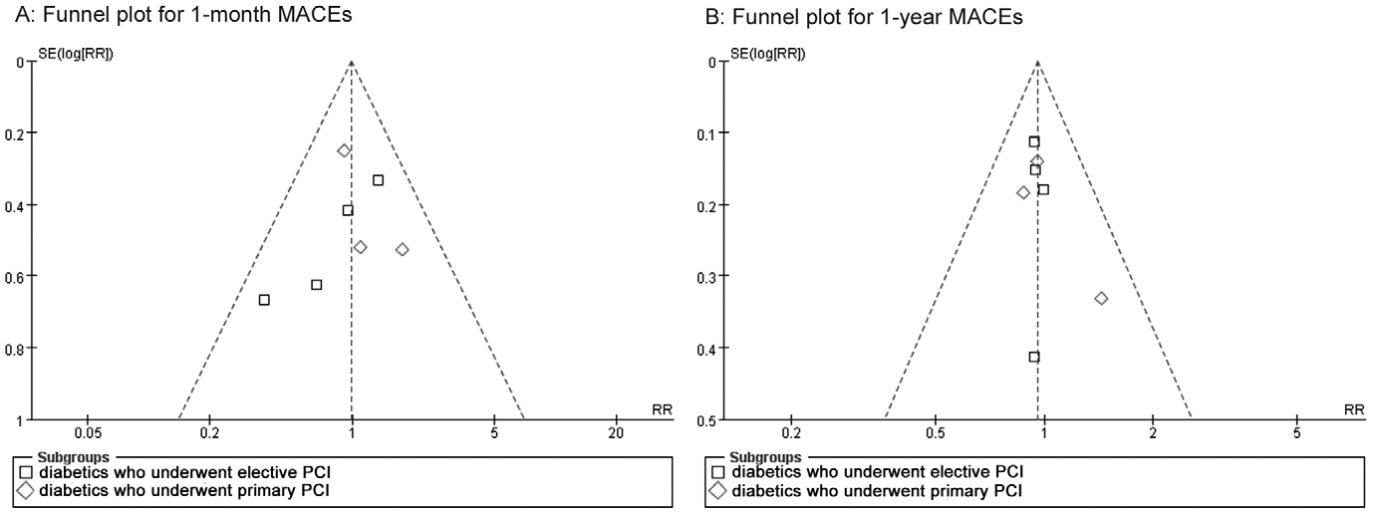
Publication bias analysis by funnel plots. Funnel plots for (A) 1-month MACEs for elective and primary PCI; (B) 1-year MACEs for elective and primary PCI.

In order to evaluate the impact of potential publication bias, the Begg's funnel plot and Egger's publication bias plot for the treatment effect of abciximab at 1-month and 1-year were applied. The results indicated that no publication bias is observed across the studies, see Figure 5.

On the other hand, a moderate level of heterogeneity was observed when evaluating the efficacy of abciximab on 1-year mortality (*I* ^2^ = 44%, *P* = 0.16), 1-year TLR (*I* ^2^ = 57%, *P* = 0.43), and angiographic restenosis (*I* ^2^ = 46%, *P* = 0.15) in primary-PCI patients. A slight level of heterogeneity was observed when evaluating the efficacy of abciximab on 1-month re-infarction (*I* ^2^ = 18%, *P* = 0.55) and 1-month MACEs (*I* ^2^ = 11%, *P* = 0.75) in elective-PCI patients, and 1-year re-infarction (*I* ^2^ = 21%, *P* = 0.96) in primary-PCI patients. No heterogeneity was observed among the remaining evaluations.

### 8. Sensitivity analyses

Sensitivity analyses were performed to further explore our findings in elective and primary-PCI patients, and the results are listed in Table 1. First, since ticlopidine is no longer used clinically, we performed sensitivity analysis to ascertain its effect attributed to the two studies, which used ticlopidine exclusively or partially [38], [42]. Second, the potential impact of loading time was assessed. And finally, the potential impact of high (600 mg clopidogrel) and low loading dose (300 mg clopidogrel or 500 mg ticlopidine) was also evaluated. The results of sensitivity analyses were consistent with our primary results.

**Table 1:** Sensitivity analyses for patients with elective and primary PCI.

Items	No. of included patients	RR (95%CI)
**ELECTIVE PCI**		
Trials with only clopidogrel loading	1396	1-month MACEs: RR = 0.93 (0.60–1.44)1-year MACEs: RR = 0.95 (0.81–1.11)
Trials with only pretreatment	1492	1-month MACEs: RR = 0.98 (0.61–1.55)1-year MACEs: RR = 0.94 (0.79–1.11)
Trials with high loading dose	1142	1-month MACEs: RR = 1.16 (0.70–1.93)1-year MACEs: RR = 0.94 (0.79–1.12)
Trials with low loading dose	604	1-month MACEs: RR = 0.50 (0.21–1.20)1-year MACEs: RR = 0.98 (0.71–1.37)
**PRIMARY PCI**		
Trials with only clopidogrel loading	720	1-month MACEs: RR = 1.04 (0.67–1.62)1-year MACEs: RR = 0.93 (0.75–1.16)
Trials with only pretreatment	904	1-month MACEs: RR = 1.05 (0.70–1.57)1-year MACEs: RR = 0.98 (0.80–1.21)
Trials with high loading dose	720	1-month MACEs: RR = 1.04 (0.67–1.62)1-year MACEs: RR = 0.93 (0.75–1.16)
Trials with low loading dose	184	1-month MACEs: RR = 1.10(0.40–3.03)1-year MACEs: RR = 1.44 (0.75–2.75)

### 9. Effect model analysis

**Figure 6:**
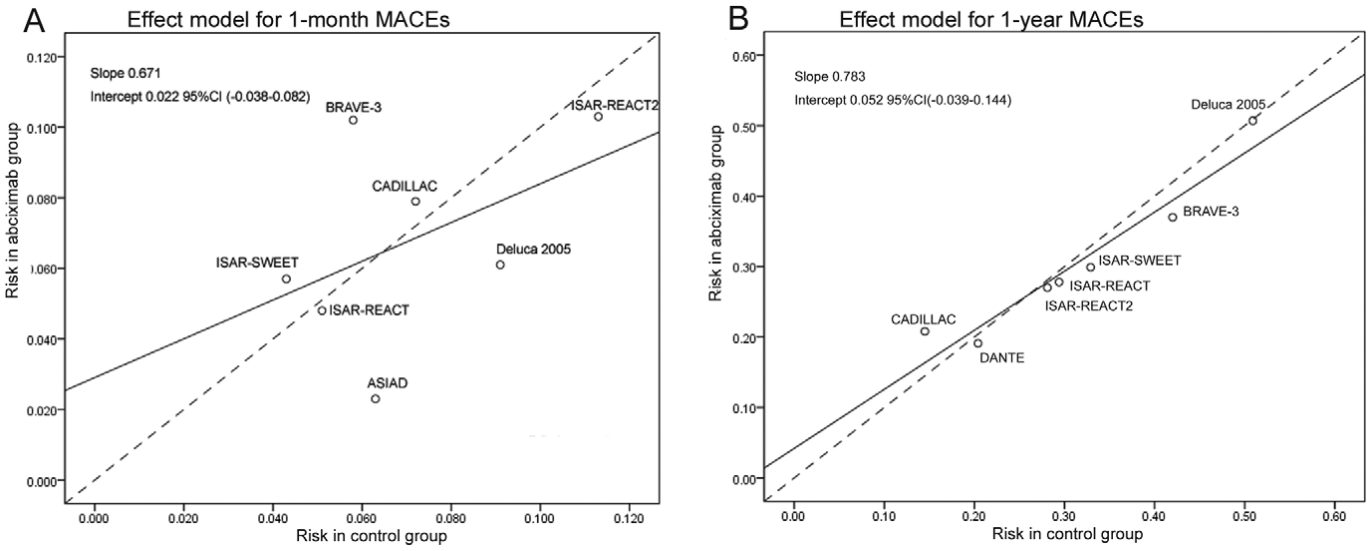
Effect model analysis of abciximab therapy on MACEs. (A) 1-month; (B) 1-year. Weight regression line (solid line) was shown between MACEs in diabetic patients who used abiximab (y axis) and non-users (x axis). The bisector (dashed line) means the lack of difference between groups.

To investigate whether the effect of abciximab in diabetic patients with thienopyridines loading is related to the incidence of MACEs in the study population, we performed an effect model analysis by using Walter weighted regression model [51] (Figure 6). It was found that the slopes of both sub-groups (including 1-month MACEs, 1-year MACEs) deviate from 1 and included the origin, indicating that the effect model was multiplicative. Overall results supported that relative risk changes of MACEs was constant regardless the incidence of events in the control group, and indicated that the effect of abciximab on MACEs in diabetic patients with thienopyridines loading might be the same in a population with a higher MACEs.

## Discussion

Although abciximab has been confirmed to improve outcomes of CAD as class II recommendation in current guidelines for elective and primary PCI [9], [11], [13], [14], [15], [21], [30], [53], [54], some recent studies demonstrated that thienopyridines loading (level I recommendation [9], [11], [21], [29], [30], [55]) would mask the benefit of glycoproteinIIb/IIIa inhibitor in these patients [34], [40], [56]. However, diabetes mellitus portents an excessive risk of thrombosis and restenosis after PCI, and several studies have proved that abiximab treatment could improve outcomes of diabetic patients undergoing PCI without thienopyridines loading [18], [20], suggesting that diabetic patients might benefit from a potent anti-platelet aggregation therapy induced by a combination of abciximab and thienopyridines. Unfortunately, our meta-analysis results from 9 trials indicated that abciximab does not reduce the occurrence of post-PCI MACEs, death, re-infarction, or angiographic restenosis in diabetic patients with either elective PCI or primary PCI after thienopyridines loading. It can even aggravate TIMI minor bleeding in the patients studied. The “effect model analysis” showed that the negative effect of abciximab was independent of the baseline risk of included population. It was worth of noting that although previous studies suggested that ACS (acute coronary syndromes) patients required a more intensive anti-platelet treatment compared to those with stable coronary diseases, our pooled results showed that abciximab did not provide additional efficacy in such patients with either elective or primary PCI. Still, the conclusion on primary-PCI patients should be interpreted cautiously, as the number of trials included in this analysis was limited.

It is possible that the conclusion could be affected by the variables such as loading dose, loading time of thienopyridine, and usage of ticlopidine. Though a 600 mg loading dose of clopidogrel achieves a more rapid platelet inhibition in comparison to the usual 300 mg dose clopidogrel or 500 mg ticlopidines [57], [58], our sensitivity analysis, stratified by applying-dose of thienopyridines, showed that no adjuvant effect of abciximab could be observed even in the low-loading group (Table 1). In another sensitivity analysis, removal of studies that used ticlopidine also did not affect the conclusion. Similarly, loading time did not appear to significantly affect the efficacy of abiciximab when we excluded the trial which did not use pretreatment.

The exact reasons for the negative results remained unknown, although one potential explanation was that the loading dose of thienopyridines could afford sufficient platelet inhibition, and there was no incremental protection achieved by the additional use of abciximab. However, this was challenged by the finding that abciximab improved outcomes in non-DM patients with NSTEMI even after thienopyridine loading (data not shown). Moreover, as GPIIb/IIIa inhibitor exerts its anti-platelet effect in a mechanism very different from clopidogrel, it appeared to be elusive why combination of the two drugs induced no synergistic effect.

Another concern involves the potential safety problems caused by combination therapy. Since both drugs could lead to post-intervention bleeding, addition of GPIIb/IIIa inhibitor to thienopyridine would possibly increase the incidence and the severity of this complication. Previous studies reported no statistically significant difference in the occurrence of major bleedings between abciximab group and control group in average patients [42], [43], [44], [59]. In our analysis, abciximab did not increase the rate of post-PCI major bleeding in diabetic patients with elective-PCI, however, the results from ISAR-REACT2 and BRAVE3 indicated that abciximab might increase the risk of major bleeding in diabetics with primary-PCI. Moreover, incidence of TIMI minor bleeding was also elevated significantly with the use of abciximab. Nonetheless, considering the limited sample size involved in the analysis of bleeding complications, further trials are needed to clarify this issue, especially in the ACS patients with primary-PCI. In addition, abciximab has been reported to reduce estimated glomerular filtration rate(eGFR)in CAD patients [60], [61]. Thus, diabetic patients, who are prone to renal lesion themselves due to the underlying disease, might be at increased risk of renal insufficiency, which would aggravate the outcomes [62], [63].

Previous meta-analysis studies had evaluated the role of GPIIb/IIIa inhibitor in diabetic patients undergoing PCI, however, patients in most trials included in the two analyses did not receive thienopyridine loading [18], [20]. Furthermore, different kinds of GPIIb/IIIa inhibitors such as tirofiban, eptifibatide, and abciximab were used in these trials. Although these three GPIIb/IIIa inhibitors all block the final common pathway of platelet aggregation by occupying the GPIIb/IIIa receptor, abciximab, tirofiban and eptifibatide differ in chemical structure, binding site and pharmacokinetics [19], [64]. It is still not clear whether abciximab has additional benefit compared to the other two “small molecule” agents (eptifibatide and tirofiban) [65], [66], [67], [68], and recent *in vitro* experiments have shown that eptifibatide produced more potent and rapid effect on platelet disaggregation than abciximab [69], suggesting heterogeneity in efficacy among different types of GPIIb/IIIa inhibitors. Furthermore, there was not enough evidence to confirm the efficacy of small molecular agent (tirofiban/eptifibatide) in PCI patients with clopidogrel loading (level of evidence is B in the latest guidelines [9], [11], [21], [22], [30], [55]). Thus, in comparison with prior meta-analysis, our study included trials applying thienopyridine loading, and abciximab was adopted with the same protocol in all included trials, and as a result, added between-study homogeneity.

The current meta-analysis has several merits. First, this is the first meta-analysis evaluating the benefits and safety of abciximab in diabetic patients with thienopyridines loading. Our results challenge the current guidelines on the usage of abciximab, which was recommended in high-risk population including diabetic patients even with thienopyridines loading. Second, we have used different search methods and posed no restriction to language in order to identify all eligible trials. Third, the included trials are of high methodological quality. And fourth, we performed sensitivity analysis to assess the validity and reliability of the primary results.

However, this meta-analysis also has its limitation in certain aspects. First, the included trials vary in sample size and intervention methods (both drug-eluting stents and bare metal stents were included), although their influence was partially alleviated by the absence of heterogeneity through χ^2^ test and I-square test. Second, the majority of patients enrolled in these trials have type 2 DM and the information on glycemic control of patients during follow-up was not available in most trials. Third, although our conclusion was based on the two types of AMI including STEMI and NSTEMI (data no shown), the theory should be assessed by more RCTs including either STEMI or NSTEMI patients separately [59]. And fourth, we should be cautious about the conclusion because of the limited sample size and number of the trials included in the analysis. Additional, multi-centered RCT is warranted to confirm this conclusion.

In conclusion, our meta-analysis suggests that abciximab does not significantly decrease the prevalence of mortality, MACEs, and angiographic restenosis at either short-term (1-month) or long-term (1-year) follow-up. On the contrary, it increased TIMI minor bleeding in diabetic patients who underwent either elective or primary PCI after thienopyridines loading. The only potential benefit was to decrease TLR rate in elective PCI at 1-year follow-up. However, further clinical trials are needed to clarify this important issue.

## Supporting Information

Table S1 Characteristics of included trials.
(DOC)

Table S2 Diabetic patient characteristics in randomized trials of adjuvant therapy with abciximab.
(DOC)

Table S3 Flow diagram.
(DOC)

Table S4 Abciximab Meta-analysis: Checklist summarising compliance with MOOSE guidelines.
(DOC)
