# Supplementary material for: Efficacy and Safety of Abciximab in Diabetic Patients Who Underwent Percutaneous Coronary Intervention with Thienopyridines Loading: A Meta-Analysis
Source: PLoS One. 2011 Jun 3;6(6):e20759. doi: 10.1371/journal.pone.0020759 (PMC3109002; doi:10.1371/journal.pone.0020759)
Supplement: Table S1 — Characteristics of included trials. (DOC) [file pone.0020759.s001.doc]

**Table S1. Characteristics of included trials**

| trial | year | location | Number | inclusion | Comparator | blind | Timing of Loading dose | Post-PCI  treatment | Follow-  up | Dephi  Criteria#(39) |
| --- | --- | --- | --- | --- | --- | --- | --- | --- | --- | --- |
| **ELECTIVE PCI** |  |  |  |  |  |  |  |  |  |  |
| **DANTE**  Chaves et all  (38) | 2004 | brazil | 96 | 1.type2 diabetes,  2.CAD exculde  AMI | control | Open-  label | Ticlopidine  500mg,  Started >24hours before PCI | Ticlopidine maintained for 4weeks aspirin indefinitely | 1year | 7 |
| **ISAR-SWEET**  Mehilli et all(39) | 2004 | European;  US | 701 | 1.DM ,  2.CAD exclude AMI | Placebo | double-blind | Clopidogrel  600 mg  at least two hours before the PCI | aspirin indefinitely;  clopidogrel 75 mg twice a day until discharge(≤3days),  followed by 75mg/d clopidogrel≥6months | 1month  1year | 9 |
| **ISAR-REACT**  Kastrati  et all(34)  Schomig  et all(40) | 2004 | European;  US; | 441 | 1.Type2 diabetes,  2.CAD exclude AMI, | Placebo | double-blind | Clopidogrel  600 mg  at least two hours before the PCI | aspirin indefinitely;  clopidogrel 75 mg twice a day until discharge(≤3days),  followed by 75mg/d clopidogrel≥1months | 1month  1year | 9 |
| **ASIAD**  Chen et all(41) | 2005 | Asia | 254 | 1.Type2 diabetes,  2.CAD  exclude  AMI | Placebo | double-blind | Clopidogrel  300 mg  at least 12 hours before the PCI | 75mg/d for 4weeks | 1month  6months | 6 |
| De luca et all  (35) | 2005 | Italy | 122 | 1.Type2 diabetes,  2.CAD  exclude  AMI | Placebo | double-blind | Clopidogrel  300 mg  Before or immediately after PCI | Clopidogrel 75mg/d for 4weeks  or ticlopidine 250mg orally twice a day  for 4weeks | 1month  6months  1year | 7 |
| Deluca  (36) | 2008 | Italy | 124 | 1.DM  2.CAD exclude AMI | Placebo | double-blind | Clopidogrel  300 mg  Before or immediately after PCI | Clopidogrel 75mg/day for nine months | 6month | 7 |
| **PRIMARY PCI** |  |  |  |  |  |  |  |  |  |  |
| **CADILLAC**  Stucky et all  (42) | 2005 | European;  US | 184 | 1.DM,  2.AMI | control | double-blind | Clopidogrel  300 mg or  Ticlopidine 500mg  Before undergoing catheterization | Aspirin 325mg/d;  Clopidogrel 75mg/d for 4weeks  or ticlopidine 250mg orally twice a day  for 4weeks | 1month  1year | 8 |
| **ISAR-REACT 2**  Kastrati(43)  Ndrepepa(44) | 2006 | European;  US | 536 | 1.DM  2.AMI  3.Early PCI <6hr | Placebo | double-blind | Clopidogrel  600 mg  at least two hours before the PCI | Clopidogrel 75mg twice a day until discharge(≤3days),  followed by 75mg/d clopidogrel≥1months | 1month  1year | 9 |
| **BRAVE3**  Mehilli et all(45)  Schulz et all(46) | 2009 | European | 184 | 1. DM 2. AMI 3. primary PCI | Placebo | double-blind | Clopidogrel  600mg  at least two hours before the PCI | Clopidogrel 75mg twice a day until discharge(≤3days),  followed by 75mg/d clopidogrel≥1months | 1month  1year | 9 |

“DM”: diabetes mellitus; “CAD”: coronary artery disease; “AMI”: acute myocardial infarction; “PCI”: percutaneous coronary intervention;

“#”:Number of Delphi criteria met out of a total of nine(39)
