# Supplementary material for: Efficacy and Safety of Abciximab in Diabetic Patients Who Underwent Percutaneous Coronary Intervention with Thienopyridines Loading: A Meta-Analysis
Source: PLoS One. 2011 Jun 3;6(6):e20759. doi: 10.1371/journal.pone.0020759 (PMC3109002; doi:10.1371/journal.pone.0020759)
Supplement: Table S2 — Diabetic patient characteristics in randomized trials of adjuvant therapy with abciximab. (DOC) [file pone.0020759.s002.doc]

**Table S2. Diabetic patient characteristics in randomized trials of adjuvant therapy with abciximab**

| Tria Trials | Age (±SD) | Male (%) | Insulin treatment (%) | Hypertension (%) | Hyperlipidemia (%) | Smokes (%) | PMI  (%) | Multivessel disease  (%) | Stenting (%) | 1-mon. MACEs (%) | 1-year  MACEs (%) |
| --- | --- | --- | --- | --- | --- | --- | --- | --- | --- | --- | --- |
| **ELECTIVE PCI** |  |  |  |  |  |  |  |  |  |  |  |
| **DANTE**  Chaves et al (32) | A: 58.9±9.8  C: 60.6±8.5 | A: 53.2  C: 44.9 | A: 19.1  C: 16.3 | A: 74.5  C: 75.5 | A: 65.9  C: 72.3 | A: 63.8  C: 53.1 | A: 67.4  C: 32.7 | A: 48.7  C: 53.7 | 100 | NA | A: 19.1  C: 20.4 |
| **ISAR-SWEET**  Mehilli et al (33) | A: 67.9±9.1  C: 67.3±9.7 | A: 73  C: 76 | A: 29  C: 28 | A: 71  C: 72 | A: 56  C: 60 | A: 15  C: 16 | A: 35  C: 33 | A: 85  C: 82 | 90 | A: 5.7  C: 5.3 | A: 29.9  C: 32.2 |
| **ISAR-REACT**  Kastrati (29)  Schomig (34) | A: 68.0±9.5  C: 67.4±9.2 | A: 74  C: 77 | A: 0  C: 0.9 | A: 60  C: 62 | A: 48  C: 50 | A: 14  C: 18 | A: 32  C: 32 | A: 80  C: 80 | 91 | A: 5  C: 5 | A: 27.8  C: 29.4 |
| **ASIAD**  Chen (35) | A: 61.6±10.4  C: 61.4±10.5 | A: 75.0  C: 72.2 | A: 10.9  C: 16.7 | A: 76.6  C: 75.4 | A: 65.6  C: 73 | NA | A: 39.8  C: 42.1 | A: 75.8  C: 68.4 | A: 94.6  C: 91.4 | A: 2.3  C: 6.3 | NA |
| Deluca 2005 (30) | 61.9± 10.1 | 65.6 | A: 42  C: 43.4 | A: 59.0  C: 65.2 | A: 46.7  C: 50.7 | A: 52.2  C: 54.7 | A: 12.3  C: 13 | A: 13  C: 11.3 | 100 | NA | A: 50.7  C: 50.9 |
| Deluca 2008  (31) | 63.1±7.4 | 62.1 | A: 38.6  C: 34.8 | A: 66.6  C: 40.9 | NA | A: 34.1  C: 36.4 | A: 12.1  C: 10.6 | A: 18.9  C: 21.2 | 100 | A: 6.1  C: 9.1 | NA |
| **PRIMARY PCI** |  |  |  |  |  |  |  |  |  |  |  |
| **CADILLAC**  Stucky (36) | A: 62  C: 60 | A: 61.4  C: 58.4 | NA | A: 68.7  C: 62.4 | A: 44.6  C: 41.6 | A: 38.6  C: 35.6 | A: 13.3  C: 13.8 | A: 15.7  C: 28.7 | A: 97.6  C: 99 | A: 7.2  C: 7.9 | A: 14.8  C: 20.9 |
| **ISAR-REACT 2**  Kastrati (37)  Ndrepepa (38) | A: 68.9±9.9  C: 68.4±10.0 | A: 69.0  C: 68.0 | A: 36.5  C: 30.3 | A: 73.0  C: 74.6 | A: 66.7  C: 61.3 | A: 17.1  C: 18.7 | A: 29.8  C: 23.2 | A: 86.1  C: 82.0 | 97.4 | A: 10.3  C: 11.3 | A: 23.3  C: 28 |
| **BRAVE3**  Mehilli et al (39)  Schulz et al (40) | A: 65.6±10.0  C: 64.5±11.3 | A: 76.5  C: 65.1 | A: 21.4  C: 17.4 | A: 82.7  C: 93.0 | A: 50.0  C: 37.2 | A: 41.8  C: 31.4 | A: 10.2  C: 15.1 | A: 77.6  C: 77.9 | 93 | A: 10.2  C: 5.8 | A: 36.7  C: 41.9 |

“A” is the diabetic patients treated with abciximab who underwent PCI; “C” is the control group.

“NA” is defined as the data is not available in the study; “PMI” previous myocardial infarction; “MACEs” major adverse cardiac events
