# Supplementary material for: Efficacy and Safety of Abciximab in Diabetic Patients Who Underwent Percutaneous Coronary Intervention with Thienopyridines Loading: A Meta-Analysis
Source: PLoS One. 2011 Jun 3;6(6):e20759. doi: 10.1371/journal.pone.0020759 (PMC3109002; doi:10.1371/journal.pone.0020759)
Supplement: Table S3 — Flow diagram. (DOC) [file pone.0020759.s003.doc]

**Table S3. Flow diagram**

**Screening**

**Included**

**Eligibility**

**Identification**

Records identified through database searching
(n = 308 )

Additional records identified through other sources
(n = 0 )

Records after duplicates removed
(n = 9 )

Records screened
(n =297 )

Records excluded
(n = 281 )

Full-text articles assessed for eligibility
(n = 16 )

Full-text articles excluded, with reasons
(n = 4 )

Studies included in qualitative synthesis
(n = 12 )

Studies included in quantitative synthesis (meta-analysis)
(n = 12 ，including 9 trials )
